# *Epilepsia Open*—June 2024 Announcements

**DOI:** 10.1002/epi4.12980

**Published:** 2024-06-03

## ILAE CONGRESSES

XIII Congreso Latinoamericano de Epilepsia

June 15, 2024 – June 18, 2024

Santo Domingo, República Dominicana

Curso Virtual sobre Epilepsia en Atención Primaria para América Latina 2024

July 8, 2024 – September 1, 2024

Online

6th ILAE School on Advanced EEG and Epilepsy in Dianalund

July 20, 2024 – July, 27 2024

Dianalund, Denmark

Advanced Pediatric Epilepsy Surgery Course 2024

August 30, 2024 – September 1, 2024

Cochin, India

15th European Epilepsy Congress

September 7, 2024 – September 11, 2024

Rome, Italy

14th International Summer School for Neuropathology and Epilepsy Surgery

September 19, 2024 – September 22, 2024

Erlangen, Germany

7th East Mediterranean Epilepsy Congress

November 14, 2024 – November 16, 2024

Manama, Bahrain

## 2025

15th Asian & Oceanian Epilepsy Congress

February 20, 2025 – February 23, 2025

New Delhi, India

36th International Epilepsy Congress

August 30, 2025 – September 3, 2025

Lisbon, Portugal

## ILAE WEBINARS

ILAE e‐Forum: Beyond the seizures in childhood onset epilepsies

June 10, 2024

Les épilepsies frontales

June 11, 2024

Status Epilepticus

June 25, 2024

Epilepsy Educational Webinar

June 28, 2024

ILAE e‐Forum: Co‐morbidities and mental health in adults with epilepsy

August 1, 2024

ILAE e‐Forum: Reducing epilepsy related mortality

September 23, 2024

ILAE e‐Forum: Optimal timing for epilepsy surgery

November 25, 2024

## OTHER CONGRESSES

SNS Annual Meeting 2024

June 6, 2024 – June 7, 2024

Basel, Switzerland

62. Jahrestagung der Deutschen Gesellschaft für Epileptologie

June 12, 2024 – June 15, 2024

Offenburg, Germany

The Comorbidities of Epilepsy

June 21, 2024

London, UK & Online

29th Korean Epilepsy Congress

June 21, 2024 – June 22, 2024

Seoul, South Korea

Basic Electroencephalography Course

June 25, 2024 – June 29, 2024

Heraklion, Greece & Online

10th Congress of the European Academy of Neurology

June 29, 2024 – July 2, 2024

Helsinki, Finland

Semiology and Neuroimaging Course 2024

July 12, 2024 – July 13, 2024

Malaysia

Pediatric Epilepsy Training Level 1: Train‐the‐trainers course 2024

July 19, 2024 – July 21, 2024

Singapore

20th Advanced San Servolo Epilepsy Course

July 21, 2024 – August 1, 2024

San Servolo (Venice), Italy

Primer Congreso Paraguayo de Epilepsia

August 1, 2024 – August 2, 2024

Asunción, Paraguay

XLVI Reunión Anual del Capítulo Mexicano de la Liga Internacional Contra la Epilepsia

August 6, 2024 – August 10, 2024

Monterrey, Nuevo León, Mexico

Curso teórico práctico de EEG y epilepsia en pediatría

August 9, 2024 – August 10, 2024

Santiago, Chile

17th Baltic Sea Summer School on Epilepsy

August 15, 2024 – September 2, 2024

Online

Advanced Pediatric Epilepsy Surgery Course 2024

August 30, 2024 – September 1, 2024

Cochin, India

10th Eilat International Educational Course: Pharmacological Treatment of Epilepsy

September 15, 2024 – September 20, 2024

Jerusalem, Israel

16th International Epilepsy Course and Colloquium

September 17, 2024 – September 20, 2024

Frankfurt am Main, Germany

6th Bologna EPIPED‐EEG Course: EEG interpretation in pediatric epilepsies

September 29, 2024 – October 3, 2024

Bologna, Italy

ASEPA‐ANZAN EEG Course 2024

October 17, 2024 – October 19, 2024

Medan, Indonesia

Epilepsy Training Course

October 18, 2024 – October 20, 2024

Douala, Cameroon

SEEG Course 2024

October 31, 2024 – November 2, 2024

Thailand

Epilepsy Society of Australia Annual Scientific Meeting 2024

November 6, 2024 – November 8, 2024

Hobart, Tasmania, Australia

AES 2024 Annual Meeting

December 6, 2024 – December 10, 2024

Los Angeles, California, USA

## 2025

International Congress on Structural Epilepsy & Symptomatic Seizures 2025

April 2, 2025 – April 4, 2025

Gothenburg, Sweden

18th Eilat Conference on New Antiepileptic Drugs and Devices

May 3, 2026 – May 6, 2026

Madrid, Spain

11th Congress of the European Academy of Neurology

June 21, 2025 – June 25, 2025

Sevilla, Spain

